# Prevalence and characteristics of benign cartilaginous tumours of the shoulder joint. An MRI-based study

**DOI:** 10.1007/s00256-023-04375-8

**Published:** 2023-06-03

**Authors:** Johannes Nikolaus Woltsche, Maria Smolle, Dieter Szolar, Marko Bergovec, Andreas Leithner

**Affiliations:** 1 Department of Orthopaedics and Trauma, Medical University of Graz, Auenbruggerplatz 5, 8036 Graz, Austria; 2 Diagnostikum Graz, Graz, Austria

**Keywords:** Enchondroma, ACT, Prevalence, Shoulder, MRI

## Abstract

**Objective:**

Enchondromas (EC) of the shoulder joint are benign intraosseous cartilage neoplasms, with atypical cartilaginous tumours (ACT) representing their intermediate counterpart. They are usually found incidentally on clinical imaging performed for other reasons. Thus far the prevalence of ECs of the shoulder has been analysed in only one study reaching a figure of 2.1%.

**Materials and methods:**

The aim of the current study was to validate this number via retrospective analysis of a 45 times larger, uniform cohort consisting of 21.550 patients who had received an MRI of the shoulder at a single radiologic centre over a time span of 13.2 years.

**Results:**

Ninety-three of 21.550 patients presented with at least one cartilaginous tumour. Four patients showed two lesions at the same time resulting in a total number of 97 cartilage tumours (89 ECs [91.8%], 8 ACTs [8.2%]). Based on the 93 patients, the overall prevalence was 0.39% for ECs and 0.04% for ACTs. Mean size of the 97 ECs/ACTs was 2.3 ± 1.5 cm; most neoplasms were located in the proximal humerus (96.9%), in the metaphysis (60.8%) and peripherally (56.7%). Of all lesions, 94 tumours (96.9%) were located in the humerus and 3 (3.1%) in the scapula.

**Conclusion:**

Frequency of EC/ACT of the shoulder joint appears to have been overestimated, with the current study revealing a prevalence of 0.43%.

**Supplementary information:**

The online version contains supplementary material available at 10.1007/s00256-023-04375-8.

## Introduction

Enchondroma (EC) is a benign central cartilaginous tumour of bone that usually occurs as a solitary lesion [1–3]. It is the second most common benign cartilaginous neoplasm of bone following osteochondroma [3]. EC has to be differentiated from atypical cartilaginous tumour (ACT), formerly known as chondrosarcoma G1, representing an intermediate form of EC [4]. Distinction between these neoplasms is important, as ACTs tend to be locally aggressive and destructive wherefore surgical treatment is recommended [5, 6]. Certain radiological markers (e.g. large lesion size, periosteal reaction, perilesional oedema and endosteal scalloping) can help distinguishing ACTs from ECs [7, 8]. The latter are most typically found in small bones of hand and foot (40–65%) [1, 9, 10], followed by femur, humerus, tibia and ribs. Regarding tumourigenesis, genetic analysis of tumour tissue has revealed that enchondromas as well as chondrosarcomas frequently show mutations of IDH1 and IDH2 (isocitrate dehydrogenase) [11, 12]. As most of lesions are asymptomatic, ECs are usually found incidentally on clinical imaging as X-ray, CT (computed tomography) and MRI (magnetic resonance imaging) performed for other reasons [9]. For example, 82% of patients with an EC of the proximal humerus suffering from shoulder pain present symptomatic due to other shoulder pathologies, with rotator cuff disease being most common [9]. Therefore, difficulties concerning estimation of the true prevalence of EC and ACT are present [9, 10]. Thus far, one study based on a small-sized cohort (*n* = 477) has analysed the prevalence of ECs around the shoulder joint, reaching a figure of 2.1% [13]. The main goal of the current study was to validate this number via retrospective analysis of a large and uniform patient cohort with MRI scans of the shoulder.

## Materials and methods

### Study design and study population

The current retrospective study is based on data from a single radiologic centre carrying out MRI scans of all body sites, besides numerous other imaging modalities.

Patients that had undergone a shoulder MRI between 01.01.2007 and 01.03.2020 were included in this retrospective analysis. In this time span, 10.043 patients had had an MRI of the left shoulder, 13.388 patients had received an MRI of the right shoulder and 1.881 patients had undergone MRI scans of both shoulder joints, leading to a total of 21.550 patients that had received at least one MRI of the shoulder.

All MRI reports of the shoulder were searched electronically for the following terms: enchondroma, (atypical) cartilaginous lesion/tumour, (atypical) chondrogenic lesion/tumour, (atypical) chondromatous lesion/tumour, (atypical) cartilage lesion/tumour, (atypical) chondroid lesion/tumour, ACT, chondrosarcoma.

**Fig. 1:**
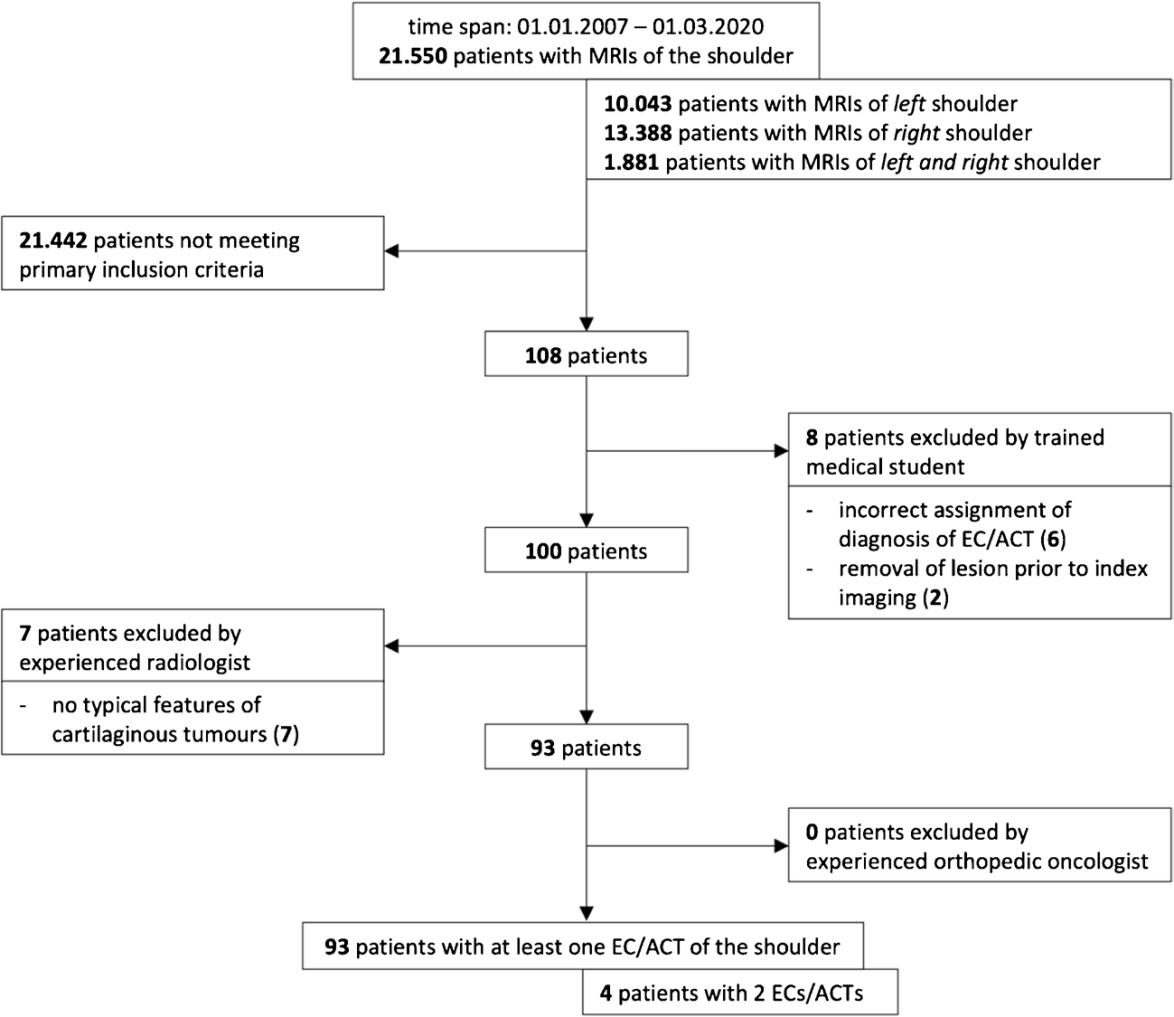
Flow chart representing the filtering of patients with a cartilaginous tumour

Altogether, 108 patients met these primary inclusion criteria and were therefore examined in further detail (Fig. 1). Eight of these patients had to be excluded for the following reasons: Three patients had been referred to the radiologic institute due to a suspected cartilaginous lesion that could not be confirmed on MRI, however; three patients presented with MRI reports not solely containing the findings of shoulder MRIs but also of other body regions and in these three patients shoulder MRIs were inconspicuous, whereas the MRIs of the other body regions revealed cartilaginous lesions; the cartilaginous neoplasms of two patients had been removed prior to index imaging.

The remaining 100 patients’ tumours were evaluated again with the aid of MRI reports and related images. Here, a definitive radiologic diagnosis of EC/ACT could be confirmed in 88 patients, while 12 patients presented with inconclusive reports and images. Therefore, an experienced senior radiologist was consulted for these cases. Thereafter, 7 of these 12 patients had to be excluded due to not showing typical features of cartilaginous tumours.

MRI-based differentiation between benign and intermediate cartilaginous tumours (i.e. ACT) was based on tumour characteristics indicative of aggressive tumour behaviour, including tumour size > 4.9 cm, periosteal reaction, perilesional oedema, and deep endosteal scalloping (≥ 2/3 of cortical thickness) [6, 8, 14]. Any chondrogenic lesion exhibiting at least one of these characteristics (*n* = 8) was thoroughly reviewed by an experienced orthopaedic oncologist, and thereafter classified as an ACT.

Overall, 93 patients received the radiological diagnosis of a cartilaginous tumour of the shoulder, with 4 patients showing 2 lesions at the same time, thereby leading to a total number of 97 tumour cases.

The study has been approved by the local ethics committee (33–630 ex 20/21).

### Lesion analysis

Cartilaginous tumours were identified as well-defined smooth/lobulated lesions within the bone marrow that presented with high signal intensity on proton-density fat-suppressed images and low signal intensity on proton-density-weighted and T1-weighted images. Subchondral lesions had to be excluded, as they might represent subchondral cysts, contusions, intraosseous ganglia, subchondral oedema, or sclerosis.

The following features were ascertained for each case: patient age (in years), patient gender (female/male), use of contrast agent (yes/no), tumour size (maximal diameter in cm), tumour site (humerus, scapula), tumour location (central/marginal; epiphysis, epimetaphysis, metaphysis, metadiaphysis, diaphysis), presence of ACT-markers (endosteal scalloping, perilesional oedema, periosteal reaction), indication for MRI (tumour-associated/other/no documented indication).

### MRI

MRI was carried out on two 3 T MRI systems with a 16-channel coil from Siemens Healthcare Diagnostics GmbH, Austria (Siemens Magnetom Skyra/Siemens Magnetom Vida). (A detailed description of the five different sequences acquired is displayed in the supplementary material section.) Clariscan 0.5 mmol/ml (gadoterate meglumine; dose 2 ml/kg body weight) was administered intravenously in 39 of 93 patients (41 of 97 tumours). This was followed by an MRI scan with “sequence 5” (coronal T1 weighted turbo spin echo with fat suppression).

### Statistics

Statistical analyses were performed using Stata Version 16.1 for Mac (*StataCorp, College Station, US*). Means and medians were provided with corresponding standard deviations and interquartile ranges (IQRs). Demographics were summarized based on the total number of patients included, whereas tumour characteristics were summarized based on total number of cartilaginous lesions found. For calculation of prevalence, diagnosis of EC/ACT per patient, and not the total number of cartilaginous lesions detected, was used. Therefore, patients without EC/ACT having undergone MRI scans of both shoulder joints during the study period were counted once only. Differences between binary (or ordinary) and continuous variables were assessed with Fisher’s exact test and t-test. A *p*-value of < 0.05 was considered statistically significant.

## Results

### Prevalence of EC/ACT in the shoulder joint

**Fig. 2:**
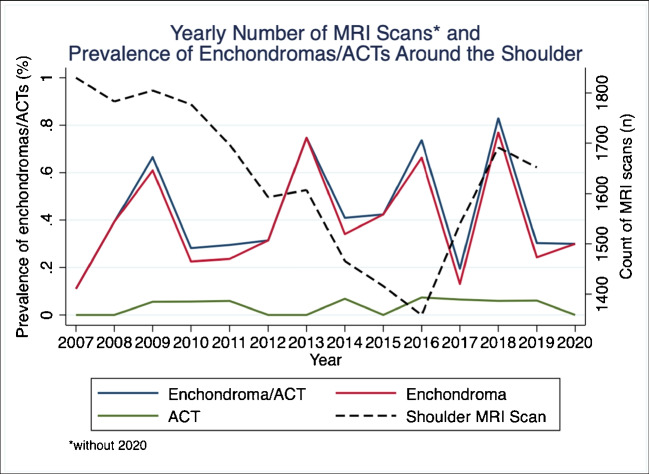
Yearly prevalence of benign cartilaginous lesions (EC + ACT), ECs and ACTs separately, as well as number of MRI scans of the shoulder joint performed

The overall prevalence of EC/ACT in the shoulder joint between 01.01.2007 and 01.03.2020 amounted to 0.43%, being 0.39% for EC and 0.04% for ACT. The yearly prevalence of benign cartilaginous lesions had an undulating but overall constant course (Fig. 2).

A cartilaginous lesion had been detected in 93 of 21.550 patients (0.43%), with four of them being diagnosed with two lesions, amounting to 97 ECs/ACTs in total. In three of four patients with two lesions, both were located in the same shoulder joint, whereas in the other patient, one cartilage lesion per shoulder joint was detected. Mean age of patients at time of MRI was 54.5 ± 11.9 years, and 56 were males (60.2%).

Reason for referral had been due to suspected cartilaginous lesion in 23 patients (24.7%), due to other pathologies in 61 patients (65.6%), and due to unknown causes in 9 patients (9.7%).

### Characteristics of EC/ACT

Of all cartilaginous lesions diagnosed, 89 and 8 were classified as EC and ACT, respectively. Mean tumour size was 2.3 ± 1.5 cm, with ACTs being significantly larger than ECs (*p* < 0.001; Table 1). Only three lesions were located in the scapula (3.1%), whilst all other ones were in the proximal humerus. The most common location within the bone was the metaphyseal area in 59 tumours (60.8%). The majority of lesions was located peripherally (*n* = 55; 56.7%). Accompanying medullary oedema was seen in 2 cases (2.1%), and in one of them being in association with the lesion itself (classified as ACT). Furthermore, endosteal scalloping was present in 7 cartilaginous tumours (Table 1), being superficial in 4 and deep in 3 cases. No periosteal reaction was observed in any case (0.0%).

**Table 1:** Characteristics of benign cartilaginous tumours detected on MRI scan, split by radiological diagnosis of EC vs. ACT (*n* = 97)

		Total Count (%)	EC (*n* = 89)	ACT (*n* = 8)	*p*-value*
Tumour Size (in cm; mean ± SD)		2.3 ± 1.5	2.0 ± 1.1	5.5 ± 1.8	< 0.001**
Side	*Left*	46 (47.4)	40 (44.9)	6 (75.0)	0.145
	*Right*	51 (52.6)	49 (55.1)	2 (25.0)	
Bone	*Proximal humerus*	94 (96.9)	86 (96.6)	8 (100.0)	0.999
	*Scapula*	3 (3.1)	3 (3.4)	0 (0.0)	
Location	*Epiphysis*	6 (6.2)	6 (6.7)	0 (0.0)	0.110
	*Epimetaphysis*	7 (7.2)	7 (7.9)	0 (0.0)	
	*Metaphysis*	59 (60.8)	56 (62.9)	3 (37.5)	
	*Metadiaphysis*	10 (10.3)	7 (7.9)	3 (37.5)	
	*Diaphysis*	12 (12.4)	10 (11.2)	2 (25.0)	
	*Scapula*	3 (3.1)	3 (3.4)	0 (0.0)	
Location in Relation to Medullary Canal	*Central*	42 (43.3)	42 (47.2)	0 (0.0)	**0.009**
	*Peripheral*	55 (56.7)	47 (52.8)	8 (100.0)	
Medullary Oedema	*No*	95 (97.9)	88 (98.9)	7 (87.5)	0.159
	*Yes*	2 (2.1)	1 (1.1)	1 (12.5)	
Endosteal Scalloping	*No*	90 (92.8)	87 (97.8)	3 (37.5)	** < 0.001**
	*Yes*	7 (7.2)	2 (2.2)	5 (62.5)	
Contrast Agent	*No*	57 (58.8)	54 (60.7)	3 (37.5)	0.268
	*Yes*	40 (41.2)	35 (39.3)	5 (62.5)	

All entries in boldface are significant, as their *p*-value is below 0.05

^*^Fisher’s exact test

^**^t-test

All tumours classified as ACT were located peripherally, whilst this was the case in 52.8% of ECs (*p* = 0.009). Also, endosteal scalloping was more often present in ACT (62.5%) as compared with EC (2.2%; *p* < 0.001; Table 1). No difference between EC and ACT was found regarding side, involved bone, location within the bone or contrast enhancement. (all *p* > 0.05; Table 1).

## Discussion

The current study was based on shoulder MRI reports of 21.550 patients who underwent imaging at a single radiology institute throughout a time span of 13.2 years. A prevalence of 0.43% for cartilaginous lesions around the shoulder joint was revealed.

As cartilaginous lesions of the shoulder joint are typically asymptomatic, their presence is mostly revealed accidentally via MRIs made for different reasons, making it hard to estimate the true prevalence of enchondromas and ACTs.

To the best of the authors’ knowledge, prevalence and characteristics of cartilage tumours in the shoulder girdle have only been examined in one small-sized study so far [13]: Hong et al. analysed 477 patients with MRI scans and reached a figure of 2.1% for the prevalence of cartilage lesions around the shoulder joint. The herein described study—based on a 45 times larger cohort (*n* = 21.550) – found a noticeably lower prevalence of 0.43%.

While Hong et al. reported on 10 patients with an enchondroma and no patient with an ACT at all [13], our study cohort included 93 patients with altogether 89 enchondromas (91.8%) and 8 suspected ACTs (8.2%). Only 8 lesions in our cohort were classified as intermediate concerning their dignity, resulting in an overall prevalence of 0.04% for ACTs in the shoulder joint. As – to the best of the authors’ knowledge – no previous study has ever focused on the prevalence of ACTs of the shoulder joint, this is the first figure elucidating this issue.

**Fig. 3:**
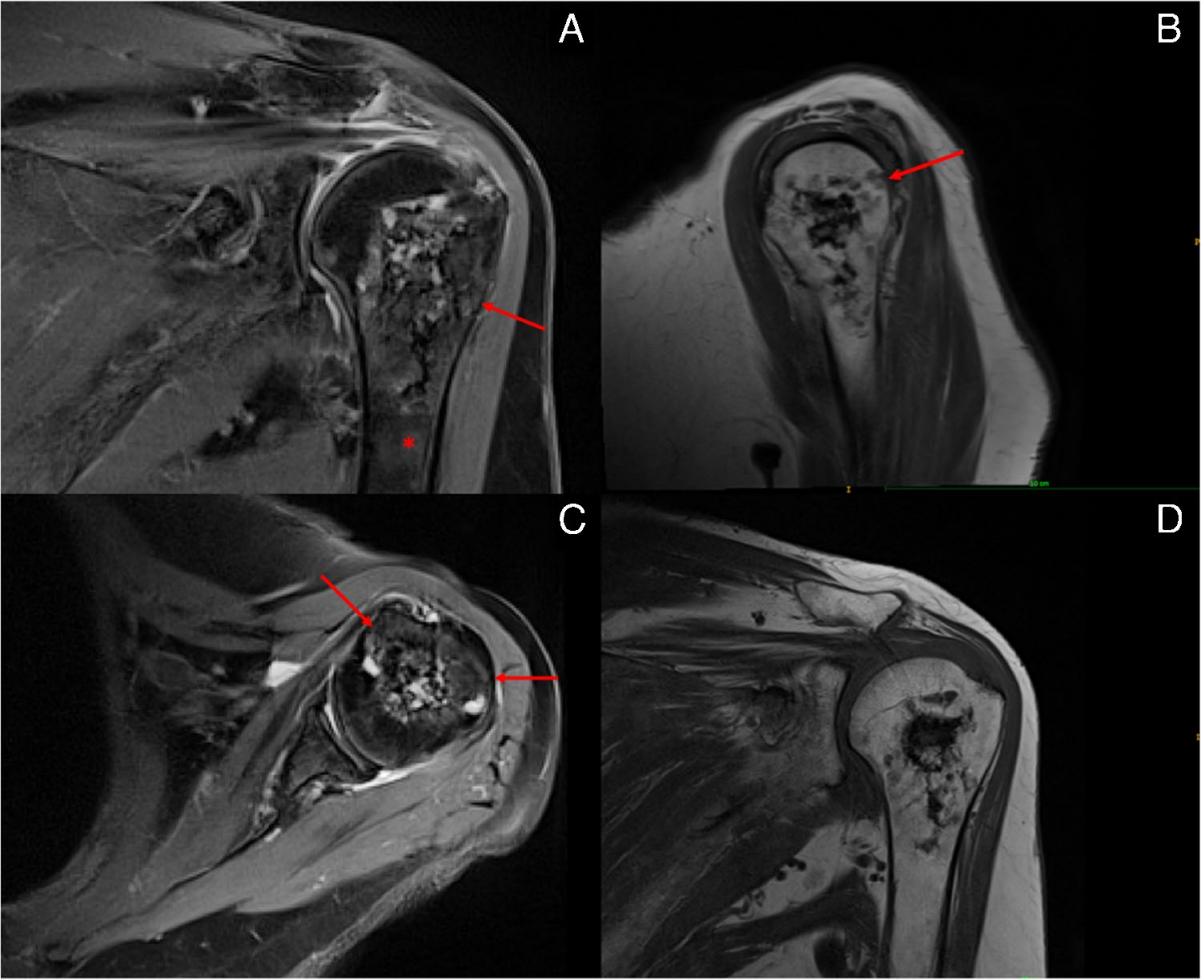
MRI scan of an ACT (maximal diameter of 5.2 cm; deep endosteal scalloping; medullary oedema) of the left humerus: **A** proton density, blade with fat suppression, coronal; **B** proton density + t2, fat suppression, dixon turbo spin echo, sagittal; **C** proton density, blade with fat suppression, transversal; **D** t1, coronal, turbo spin echo. The arrows point at endosteal scalloping, whereas the asterisk highlights medullary edema

Two different research groups have shown that the frequency of chondrosarcomas diagnosed – and especially the one of ACTs – has increased over the last decades, most likely due to intensified MRI screening, causing a rising number of accidental findings [4, 15]. Our study has not been able to validate these results for ACTs around the shoulder joint since only 8 patients presented with an ACT like lesion, too few to identify a trend (Fig. 3).

Furthermore, Davies et al. showed that 85% more patients with an enchondroma were referred to an UK-based specialist orthopaedic oncology unit between 2009–2018 than in the time span of 1999–2008, indicating a rise in incidence of enchondromas over the last decades [4]. Notably, the current study, performed between 2007 and 2020, could not confirm an increase over this time span as far as the prevalence of enchondromas of the shoulder joint is concerned.

Analysis of tumour characteristics showed that ACTs (mean size 5.5 cm) were significantly (*p* < 0.001) larger than enchondromas (mean size 2.0 cm), thereby confirming previous results by Kendell et al. who analysed intraosseous cartilaginous lesions of the fibula and highlighted the importance of lesion size as a marker of differentiation between benign and intermediate cartilaginous tumours. They found that lesions smaller than 4 cm tended to be enchondromas, whereas a size of more than 4 cm was indicative of an atypical cartilaginous tumour (formerly known as low-grade chondrosarcoma) [16].

**Fig. 4:**
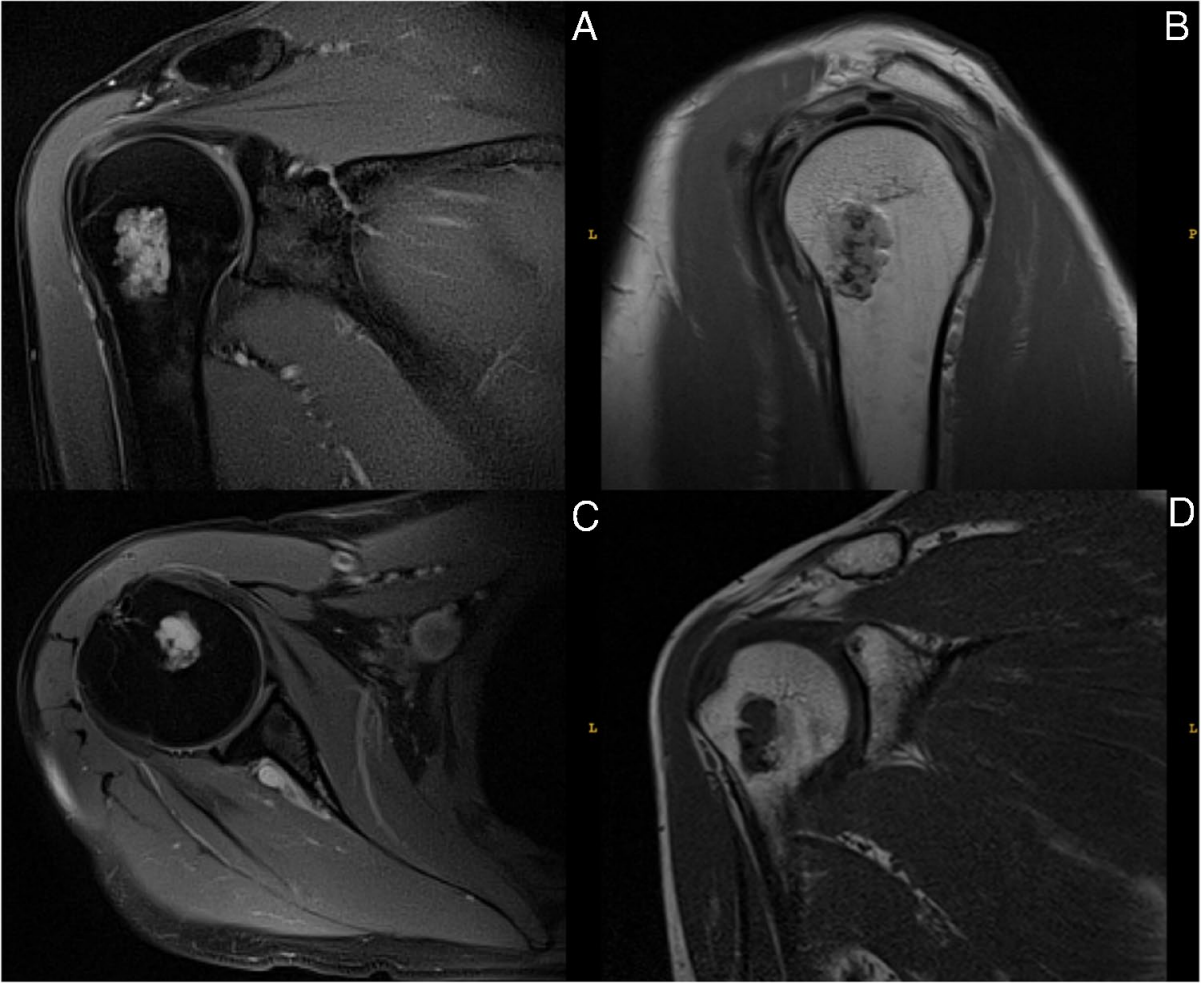
MRI scan of an enchondroma of the right humerus: **A** proton density, blade with fat suppression, coronal; **B** proton density + t2, sagittal; **C** proton density, blade with fat suppression, transversal; **D** t1, coronal, spin echo

The majority of enchondromas of the shoulder presented were located in the proximal humerus (96.6%) (Fig. 4), whereas only three ECs were found in the scapula (3.4%). In literature, the scapula is referred to as an unusual localization of enchondroma with only two retrospective studies [13, 17] and a few case reports [18–20] having published the finding of an EC at this specific anatomical site (to the best of the authors’ knowledge, altogether eight ECs of the scapula have been reported in literature up to now). Song et al. have only recently evaluated tumours of the scapula and found that among 108 cases of benign and malignant lesions of the scapula, four patients (3.7%) presented with an enchondroma [17] indicating that enchondroma represents a rare but relevant differential diagnosis when dealing with tumours of the scapula. Hong et al. discovered that 10% of shoulder enchondromas reside in the scapula. Authors of that study, however, were only able to analyse tumour characteristics of 10 cartilaginous shoulder lesions with one of them being located in the scapula [13]. As the current study was based on 97 ECs/ACTs of the shoulder joint and implicates a lower frequency for scapular ECs (3.4%), it can be assumed that the figure of Hong et al. on this matter (10%) is overestimated, as their study was based on a significantly smaller cohort [13].

There was a significant difference between ECs and ACTs (*p* = 0.009) regarding location in relation to medullary canal, with ACTs showing eccentric location significantly more often (100%) than ECs (52.8%). While in a cohort by Bui et al., reporting on 11 patients with eccentric ECs of long bones all tumours (100%) all were associated with cortical scalloping [21], the current data cannot replicate these results, as only 2 of 47 (4.3%) eccentrically located ECs presented with superficial scalloping. A major difference between the studies is represented by the fact that Bui et al. only examined eccentric lesions of long bones around the knee joint (10 tumours of the femur and 1 of the tibia) [21].

Regarding location within long bones, ECs represent intramedullary lesions typically residing in the metaphysis of long bones [10]. The theory that ECs arise out of physeal rests, which were trapped in the metaphysis of growing long bones, is mainly based on this finding [1]. However, this widely believed theory has been challenged by Douis et al. [22], who did not find any displaced cartilage in the metaphysis of long bones in skeletally immature individuals. However, ECs cannot solely be found in the metaphysis and diaphysis, with Potter et al. [23] reporting that 6.7% of patients with an EC affecting long bones (33 out of 508 patients) showed a benign cartilaginous tumour originating from the epiphysis, with the majority of these lesions residing in the proximal humerus (30%). Hong et al. discovered in their tumour cohort that even more ECs of the proximal humerus were located in the epiphysis (55.6%) than in the metaphysis (44.4%) [13]. This finding cannot be supported by our data, suggesting that only a minority of ECs (6.7%) are located in the epiphysis, thereby confirming the finding of Potter et al. for the frequency of epiphyseal location in benign intraosseous tumours of long bones [23].

Some limitations have to be considered when interpreting the results: First, diagnosis of all cartilaginous lesions was MRI-based only. Radiological criteria to differentiate enchondromas from atypical chondromatous tumours are not utterly defined, though. Second, due to the benign nature of ECs, the vast majority of patients did not undergo biopsy, wherefore histopathological assessment was not part of the diagnostic approach. Considering that histological differentiation between EC and ACT depends on the quality of the biopsy and may be prone to sampling error, it can be assumed that additional histological examination would not have significantly altered results obtained [24]. Two further studies have confirmed that histological differentiation of ECs and ACTs represents a difficult task for pathologists, as high interobserver variability in diagnosis as well as grading of cartilaginous lesions could be detected [25, 26]. Flemming et al. in 2000 and Eefting et al. in 2007 proposed a diagnostic approach that combines radiological and histological assessment for differentiation of ECs and ACTs, as pathohistological examination solely leads to unreliable diagnoses [1, 26]. Miwa et al., however, discovered that comprehensive assessment of radiological examinations solely shows high accuracy for evaluation of dignity in chondromatous lesions [27]. Furthermore, bioptic validation of conclusive radiological diagnosis of a benign intraosseous chondromatous lesion represents an unnecessary burden for a patient, which is why the majority of ECs do not undergo histological examination. Therefore, it is hard to identify a cohort of patients with histopathologically confirmed ECs, and it appears that authors of an epidemiological study with the ambition to find the prevalence of ECs and ACTs have to accept that such a study either must be based on a small cohort, where all tumours can realistically be histologically confirmed or they must accept imaging-derived diagnosis of lesions with the major advantage that a high patient number can be included. As imaging-based diagnosis has proven to do very well, even in distinguishing ECs from ACTs [27], and as significant epidemiological studies subsist on patient cohorts as large as possible, it can be assumed that a study with the ambition of finding the true prevalence of ECs and ACTs has to do without histology.

Due to the criteria chosen to differentiate between EC and ACT, statistically significant results with regards to features as tumour size emerged (Table 1). Yet, also literature [6, 8, 14] confirms that these features vary between the two cartilaginous neoplasms. Another limitation of this study is presented by the retrospective study design, as MRIs had usually been performed for other shoulder pathologies, with the incidental finding of cartilage tumours.

In summary, the herein presented large-sized study revealed an overall prevalence of 0.43% for benign and intermediate chondromatous lesions around the shoulder joint, with 8.2% of these lesions exhibiting at least one MRI feature highly suspicious of ACT. Prevalence of ECs around the shoulder joint has probably been slightly overestimated in the past, however, as 1 in 233 patients will show an enchondroma in an MRI of the shoulder, it remains an important differential diagnosis not to be mistaken for other pathologic entities.

### Supplementary information

Below is the link to the electronic supplementary material.

Supplementary file1 (DOCX 16 KB) Supplementary Table. Technical description of the shoulder MRI sequences

## Data Availability

The datasets used and/or analysed during the current study are available from the corresponding author on reasonable request.
