# Supplementary material for: Prevalence and characteristics of benign cartilaginous tumours of the shoulder joint. An MRI-based study
Source: Skeletal Radiol. 2023 Jun 3;53(1):59–66. doi: 10.1007/s00256-023-04375-8 (PMC10661778; doi:10.1007/s00256-023-04375-8)
Supplement: Supplementary file 1 — Supplementary file1 (DOCX 16 KB) Supplementary Table. Technical description of the shoulder MRI sequences [file 256_2023_4375_MOESM1_ESM.docx]

|  | **Sequence 1** | **Sequence 2** | **Sequence 3** | **Sequence 4** | **Sequence 5** |
| --- | --- | --- | --- | --- | --- |
| **Section** | coronal | sagittal | transversal | coronal | coronal |
| **Weighting** | PD* | PD and T2 TSE**/  PD dixon TSE | PD blade | T1 TSE | T1 TSE |
| **Fat suppression** | yes | no | yes | no | yes |
| **Field of view (mm)** | 160/150 | 160/130 | 160/140 | 150/140 | 150/140 |
| **Matrix** | 320x320/256x256 | 384x384/272x320 | 256x256/256x256 | 403x448/269x384 | 256x320/269x384 |
| **Repetition time (ms)** | 2250/2250 | 3520/2490 | 2250/3120 | 574/598 | 660/550 |
| **Echo time (ms)** | 46/49 | 28/37 | 43/50 | 12/11 | 11/11 |
| **Slice thickness (mm)** | 3/3 | 3/3 | 3/3.5 | 3/3 | 3/3 |
| **Interslice gap (mm)** | 0.3/0.9 | 0.9/0.6 | 0.3/1 | 0.3/0.9 | 0.9/0.9 |
| *PD = proton density  **TSE = turbo spin echo | | | | | |
